# Predictors of Self-Reported Health Status of Ready-made Garment Workers in Bangladesh in Post Global Economic Recession Period

**DOI:** 10.7759/cureus.5742

**Published:** 2019-09-24

**Authors:** Taufique Joarder, Shafiun N Shimul, Md Imran Hasan, Farjana Yeasmin, Anwar Islam

**Affiliations:** 1 Epidemiology and Public Health, FHI 360 Bangladesh, Dhaka, BGD; 2 Miscellaneous, Institute of Health Economics, University of Dhaka, Dhaka, BGD; 3 Epidemiology and Public Health, Institute of Public Health Nutrition, Dhaka, BGD; 4 Radiology, Bangladesh Institute of Research and Rehabilitation In Diabetes, Endocrine and Metabolic Disorders, Dhaka, BGD; 5 Epidemiology and Public Health, School for International Development and Global Studies, University of Ottawa, Ottawa, CAN

**Keywords:** economic recession, ready-made garments, self-reported health status, predictors, bangladesh

## Abstract

Introduction

There has been disagreement within academia in Bangladesh on whether the global economic recession of 2008-2009 came out as a bane or a boon to their economy and for their people, particularly workers in the ready-made garments (RMG) sector; therefore, we sought to conduct a study among currently employed and recently unemployed RMG workers to examine the influence of recession on their self-reported health status.

Methods

This cross-sectional study was conducted among 200 workers across 20 factories and 108 recently unemployed workers from different locations of Dhaka. Workers were selected based on a systematic sampling method from 20 randomly selected factories. Unemployed respondents were selected via snowball sampling. A questionnaire was prepared to cover different socio-demographic variables, which were then explored against an outcome variable of how the respondents rate their current health status (2009) compared with their past health status during the economic recession period (2008). A simple logistic regression was conducted for each of the independent variables with the outcome variable. Finally, all independent variables were loaded against the outcome variable, and multiple logistic regression was run.

Results

The only statistically significant predictor of self-reported health status was age, which indicated a 4% decrease (p = 0.05; 95% confidence interval (CI), 0.9203417 to 1.000015) in improved or better health with each year increase in age, holding other variables constant. Respondent health status was unchanged or even improved after the period of recession. The employed group had 1542.061 Taka (approximately $20) more average monthly family income than the unemployed group (two-sample t-test p-value 0.007), their health status was not affected (odds ratio (OR) 0.998; p-value 0.907).

Conclusion

The absence of an association between self-reported health status and economic recession is not uncommon, and explanations have been proposed for this phenomenon.

## Introduction

As land for agricultural usage is exponentially diminishing, Bangladesh’ historical identity as an agrarian society is being challenged. Labor-intensive industries, such as the ready-made garments (RMG) sector, are thriving based on the availability of cheap labor, which is significantly contributing to the economy of the country [1-2]. According to the International Standard Classification of the United Nations, the RMG sector is defined as “those establishments which cut and/or stitch/make-up garments out of woven or knitted fabrics without being involved in the manufacture of fabrics”[1]. Before the liberation of Bangladesh from Pakistan in 1971, there was only one garment factory in then-East Pakistan. However, after their independence, the number of RMG factories witnessed a steady increase to eight in 1977; to 134 in 1984; to 2,353 in 1996; and 4,740 by 2008 [2]. RMG is the chief earning source through export for Bangladesh, accounting for 75% of total export earnings and 13% of the total gross domestic product; there are approximately 3.6 million garment workers, among whom more than 80% are women [3]. Although decreased from 15.9% growth in the 2007-2008 fiscal year to 10.3% growth in the 2008-2009 fiscal year, RMG exports to the world market remained positive, even during the period of recession [4].

The effects of the global economic recession have deepened and broadened its adverse impact across countries [5]. Economic downturns converted into recession in the developed nations, and its repercussions hurt the developing and least-developed countries as well. Economic recessions tend to hurt all sectors of the economy; however, the poor and working-class face more negative consequences due to the economic downturn. The International Labour Organization forecast that 1.07 million people worldwide would be unemployed during the period of recession in 2008 [6].

However, in the context of Bangladesh, it was difficult to determine the recessionary impact, and concerned professionals and academia were divided on this issue. For example, garment manufacturers demanded government subsidies [7], while a group of academicians shunned the idea, claiming that the recession actually came as a boon rather than a bane [5,8]. Those who opposed the impact of recession argued that most of the economic and social indicators were still in favor of Bangladesh, which might be explained by two factors. First, the exports of Bangladesh are mostly consumer goods whose demand does not fall sharply as income goes down. Second, Bangladesh rather improved its competitive position in the world apparel market. It was also argued that the decrease in income in developed countries might have increased the demand for Bangladeshi RMG as most RMG products of Bangladesh are of lower price [4].

On the contrary, the advocates of recessionary impact argued that the export sector was at massive risk due to two important reasons. First, Bangladeshi exports are too RMG-dependent (approximately 70%). Second, most exports are to the United States of America and European Union countries, and most of those nations were facing massive economic recession, which might decrease the demand for RMG goods in those countries and thus might exert a negative effect on the export earnings of Bangladesh. According to Bangladesh Garment Manufacturers and Exporters Association (BGMEA) statistics, the growth record of woven garments exports in July 2008 was approximately 58%, as compared to only 5.38% in April 2009. For the knitwear sector, growth was approximately 84.70% in July 2008, but only 0.14% in April 2009. BGMEA warned that growth would decline to zero or even a negative percentage if proper government support were not ensured.

Moreover, if infrastructure problems persisted, Bangladesh could lose its competitive position in the long term. Besides the risk of lower demand for RMG, there were other related risks [9]. First, there was a fear of not getting money from importers if any large firm defaulted. Second, there was an increased chance of deferred payment. Third, importers could take advantage of the economic crisis by reducing prices through favorable negotiations. Therefore, some positive and some negative forces were likely to work in the RMG exports sector, and the net impact would determine the final outcome and employment in this sector.

Postulating that economic recession could impact the health of RMG workers, we conducted this study to identify the predictors of self-reported health status among RMG workers in Bangladesh in the 2009 post-global economic recession period. We considered employment status to be a surrogate for the economic recession at the micro or individual level. Therefore, we were particularly interested to see if employment status was associated with self-reported health status. Other socio-demographic variables were also considered to see if there was any association with those factors, and also to control for those factors while evaluating employment status.

## Materials and methods

We conducted a cross-sectional study in 2009 among two groups: RMG employees and recently unemployed workers from RMG factories. We randomly selected 20 garment factories from in and around Dhaka, using a list developed by Building Resources Across Communities (BRAC), a Bangladeshi non-government organization. From each factory, 10 employees were selected using systematic random sampling in each factory. Data collection from the unemployed group was the most difficult part, as the factories from where they were terminated did not have complete information. In other factories where there was some contact information, factory representatives were reluctant to provide it. So, data collectors collected information from other employees in regions where most garment workers lived. They targeted recently unemployed persons using snowball sampling. Data were collected through face-to-face interviews conducted using a structured questionnaire. A research team consisting of three public health researchers developed the questionnaire and pre-tested it with five respondents from one garment factory at Narayanganj (a city near Dhaka). The total number of respondents was 308, which included 200 RMG workers and 108 unemployed workers.

To explore our outcome variable of self-reported health status compared to the recession period, we posed the following question: “In general, how would you rate your current health when comparing it to your past health status (e.g., one year to six months ago)?” Initially, we designed the variable as a categorical one and gathered the responses coded as “much better, better, similar, a little worse, and much worse.” Because the objective of our analysis was to see whether the health status worsened or improved, we dichotomized the responses into “worse than the recession period” and “improved or similar to the recession period.” The following independent variables were explored: employment status, age (in years), gender, marital status (dichotomized into currently married and currently not married), number of family members, years of education, and average family income (in Bangladeshi taka per month). 

An exploratory data analysis was conducted to understand the important characteristics of the variables used in the data set. For dichotomous variables, 2x2 tables were compared against the outcome variable, and a Fisher’s exact test was conducted. For continuous variables, mean and standard deviations were explored. Collinearity was tested for the independent variables, and the variance inflation factor (VIF) values were found to be reasonably low. Mean VIF value was 1.38, with the highest value being only 1.57. A simple logistic regression was conducted for each of the independent variables with the outcome variable to better understand the relationships between them. Then, all independent variables were loaded against the outcome variable, and multiple logistic regression was run. Based on the backward method of model building, gender and marital status were removed from the model. The new model was considered as the null model, and a likelihood ratio test was conducted against the previous model, which was considered as the extended model. With the p-value being insignificant (0.8060), we decided to adopt the more parsimonious model, which included the following variables: employment status, age, number of family members, years of education, and average family income. Hosmer-Lemeshow goodness of fit test was conducted to check whether the model fits the data. All statistical analyses were conducted using Stata version 11.0 (StataCorp, LLC; College Station, TX, USA)

Ethical principles were strictly adhered to during the entire research process. Approval was obtained from the Ethical Review Committee of the James P. Grant School of Public Health, BRAC University.

## Results

Of 308 respondents, 200 were employed RMG workers, and 108 were recently unemployed (within the last six months) RMG workers. Among the employed respondents, 82% were female, which is similar to the national estimate of the gender distribution of garment workers. The mean age of respondents was 24 years. The mean age of respondents with improved or similar health was 23 years, and the mean age of those with deteriorated health was 26 years. Gender distribution among the improved or similar health groups and the deteriorated health group was similar (Table 1).

**Table 1:** Demographic characteristics by self-reported health status

Variable	Improved or similar health status group	Deteriorated health status group	Total
Age in years (mean, SD)	23.35 (5.42)	25.58 (8.35)	24.03 (6.52)
Gender (number, %)			
Male	45 (68.18)	21 (31.82)	66 (100)
Female	169 (69.83)	73 (30.17)	242 (100)
Marital status			
Married	116 (66.67)	58 (33.33)	174 (100)
Not married	98 (73.13)	36 (26.87)	134 (100)
Number of family members (mean, SD)	3.47 (1.82)	3.97 (2.17)	3.62 (1.94)
Education in years (mean, SD)	6.45 (2.67)	6.51 (2.81)	6.46 (2.71)
Average household income per month in taka (mean, SD)	8360.84 (4218.14)	8541.53 (5889.19)	8416.112 (4782.17)

The number of family members was higher in the deteriorated health group. In fact, the number of family members was one of the independent variables significantly associated with self-reported health status in the simple logistic regression involving these two variables. Results demonstrated a 12% reduction (p = 0.04) in improved or similar health status with an increase of one additional member in the family. Level of education and monthly household income were similar in both the improved and deteriorated health groups.

Employment status was the most important variable for our analysis, as it was considered a surrogate for the effect of economic recession. Therefore, lower health status among the unemployed group would indicate that the recession had an impact on health status. Due to the importance of employment status in the context of our research objective, we conducted a separate analysis with this variable. However, the employed group had 1542.061 taka (approximately $20) more average monthly family income than the unemployed group (two-sample t-test; p = 0.007), and their health status was not affected (odds ratio [OR], 0.998; p = 0.907). Since the OR is almost 1.0, it implies that the unemployed group does not have worse self-reported health status than the employed group. Cross-tabulation with a Fisher’s exact test also demonstrated no difference between employed and unemployed groups in terms of health status (Table 2). It shows both employed and unemployed groups have 69% of respondents with improved health and 31% with deteriorated health, respectively. A Fisher’s exact test p-value of 1.00 reinforces the evidence of a lack of difference between employed and unemployed groups.

**Table 2:** Cross-tabulation of employment status against health status Fisher’s exact test p-value: 1.00

	Employment status		
Health status	Employed (number, %)	Unemployed (number, %)	Total (number, %)
Improved	139 (69.50)	75 (69.44)	214 (69.48)
Deteriorated	61 (30.50)	33 (30.56)	94 (30.52)
Total (number, %)	200 (100)	108 (100)	308 (100)

Finally, our multiple logistic regression analysis proves that neither employment status nor other variables such as the number of family members, years of education, and average family income had an association with the improved self-reported health status (Table 3). The only variable that was marginally significantly associated with the outcome variable was age. The analysis shows that improved health status was decreased by 4% (OR, 0.96; p = 0.05; CI, 0.92 to 1.0) with every one-year increase in age, adjusting for other variables (employment status, number of family members, years of education, and average monthly family income). RMG factory employees were 14% more likely to have improved health status, controlling for other variables; however, the association was not statistically significant (OR, 1.14; p = 0.66; CI, 0.64 to 2.03). Other variables were also not significantly associated with health status improvement.

**Table 3:** Result of multiple logistic regression analysis with improved self-reported health status as the outcome variable Pseudo R2 = 0.02 Hosmer-Lemeshow Chi2 = 4.58 (p-value: 0.80)

	Improved self-reported health status		
Independent variables	Odds ratio	p-value	95% confidence interval
Employment status	1.14	0.66	0.64 – 2.03
Age	0.96	0.50	0.92 – 1.0
Number of family members	0.90	0.13	0.78 – 1.03
Years of education	1.0	0.94	0.90 – 1.10
Average family income	1.0	0.93	0.99 – 1.00

## Discussion

To comprehend the findings of this study, it is imperative to understand two issues. First, whether there was any impact of the recession in Bangladesh, particularly on the RMG sector. Second, whether the health status of RMG workers was affected by the recession. The first issue is beyond the scope of our study and will need to be explored through macroeconomic analysis. Economists in Bangladesh were divided in their opinion on whether Bangladesh was affected by the global economic recession. Those who were proponents of the viewpoint that Bangladesh remained unaffected by the recession had three major arguments: (1) The economy of Bangladesh was not well connected with the global economy. So, the impulse of the global economic recession was not effectively transmitted in the local economy of Bangladesh [8]. (2) The economy of Bangladesh was largely dependent on foreign aid, and most of that aid had long-term commitments from donors. So, this might not affect Bangladesh’s economy in the short term. (3) The economic policy of Bangladesh was resilient enough to repel the impulse of recession and rebuild the loss quickly [4].

The other group of economists remains confident about the adverse effects of the recession on Bangladesh’s economy [10]. They presented convincing evidence against the arguments mentioned above. A study carried out by the Center for Policy Dialogue, a renowned economic research organization based in Bangladesh, argued that 85% of exports are destined for developed countries. Many expatriate workers are working in various countries and sending remittance. So, the argument that the economy of Bangladesh is not well connected with the global economy might have been true during the economic recession of 1998-1999 but was not true during the recession of 2008-2009. RMG exports were found to be slowing down in 2009 compared to previous years. The same study reported the closing of several RMG factories. All these factors indicate the adverse effect of the global economic recession on the economy of Bangladesh, especially on the RMG sector [10]. However, one sub-group of those who believe that the global recession had an impact on Bangladesh's economy remain skeptical about the effect of the recession on the RMG sector in particular. They imply that, although the country was hit by a recession, the RMG sector fared well as a consequence of it. In addition, Bangladesh is principally an exporter of mid- or low-quality RMG products, the demand for which eventually increased among RMG importer countries [8]. However, even if the RMG industry was not affected by the recession, workers were likely affected due to the impact of the recession on other aspects of their economic life, (e.g., inflation, increased price of commodities, low public health expenditure by government, and unavailability of jobs in other sectors).

The second and final concept that we need to consider is whether this economic downturn had an impact on the health of RMG workers. We concluded that RMG workers were affected by the global economic recession either directly (due to the RMG being affected) or indirectly (due to other sectors being affected). This conclusion was further supported by the work of Kwon et al. [11]. Our interview with employed and unemployed groups revealed significantly lower average monthly household income among RMG workers who lost their job within the last six months. The period of their job loss corresponds with the period of initiation of the global recession in 2008. Therefore, it can be assumed that they lost their jobs as a consequence of the global recession, and their average monthly household income also plummeted as a consequence. However, it is interesting to find that this reduction of income did not affect their health status.

However strange the non-association between economic recession and self-reported health status may seem, this phenomenon has been observed in many other studies. Due to the non-intuitive nature of these findings, those studies were not previously taken seriously; however, with consistent findings over the years, scientists are increasingly becoming curious [12-13]. Explanations for this apparently paradoxical finding have also been proposed by scientists. They argue that increased leisure time helped unemployed workers build social cohesion [14], allowed them more time with their family [15], and reduced many work-related stressors [16]. Health gain might also be attributed to reduced smoking, alcohol use, and overeating [17].

In addition to these explanations, in the context of Bangladesh, the following explanations may also be proposed. First, the study was carried out right after the period of recession. This short lapse of time might have been inadequate to reflect any change in health status. Second, both the employed and the unemployed groups belonged to a low-income group of Bangladeshis, who usually seek care from informal healthcare providers [18] or government healthcare facilities. In Bangladesh, government health facilities are free of cost. So, even if unemployed persons had a drop in their income, both groups embraced the same consequences in terms of seeking healthcare. Third, Bangladesh still has a joint family norm, as well as strong social and family ties. When someone is sick, it is usually the neighbors and the relatives who come forward to seek treatment for the patient. Borrowing money (free of interest) is an important source of meeting healthcare expenditure. When examining Australian society during the period of the Great Depression, Potts showed how greater social cohesion was observed during this period of social stress [19].

Recent unemployment status, whether as a consequence of global recession or not, did not affect the health status of RMG workers. However, more in-depth, larger, and longer studies are exceedingly warranted for the comprehensive understanding of the impact of the recession on the health of RMG workers. The International Centre for Diarrheal Disease Research, Bangladesh has a demographic surveillance system in the Matlab sub-district, which has been collecting and storing demographic data since 1966 [20]. Longitudinal data collected by them can be analyzed to examine whether there has been any difference in health status at the population level or among a specific group (e.g., RMG workers or any other group) between the pre- and post-recession period.

## Conclusions

Age was determined to be a predictor of health status in our study, and further studies should be carried out to identify other potential predictors of health status. Policymakers should consider developing a mechanism to monitor economic data, both at the macro and micro levels, and to match it with health outcomes, to identify threats to population health and to quickly take any necessary action.
